# Meiotic Transmission of *Drosophila pseudoobscura* Chromosomal Arrangements

**DOI:** 10.1371/journal.pone.0000530

**Published:** 2007-06-13

**Authors:** Richard P. Meisel, Stephen W. Schaeffer

**Affiliations:** Intercollege Graduate Program in Genetics, Institute of Molecular Evolutionary Genetics, and Department of Biology, The Pennsylvania State University, University Park, Pennsylvania, United States of America; Duke University, United States of America

## Abstract

*Drosophila pseudoobscura* harbors a rich gene arrangement polymorphism on the third chromosome generated by a series of overlapping paracentric inversions. The arrangements suppress recombination in heterokaryotypic individuals, which allows for the selective maintenance of coadapted gene complexes. Previous mapping experiments used to determine the degree to which recombination is suppressed in gene arrangement heterozygotes produced non-recombinant progeny in non-Mendelian ratios. The deviations from Mendelian expectations could be the result of viability differences between wild and mutant chromosomes, meiotic drive because of achiasmate pairing of homologues in heterokaryotypic females during meiosis, or a combination of both mechanisms. The possibility that the frequencies of the chromosomal arrangements in natural populations are affected by mechanisms other than adaptive selection led us to consider these hypotheses. We performed reciprocal crosses involving both heterozygous males and females to determine if the frequency of the non-recombinant progeny deviates significantly from Mendelian expectations and if the frequencies deviate between reciprocal crosses. We failed to observe non-Mendelian ratios in multiple crosses, and the frequency of the non-recombinant classes differed in only one of five pairs of reciprocal crosses despite sufficient power to detect these differences in all crosses. Our results indicate that deviations from Mendelian expectations in recombination experiments involving the *D. pseudoobscura* inversion system are most likely due to fitness differences of gene arrangement karyotypes in different environments.

## Introduction

**Figure 1:**
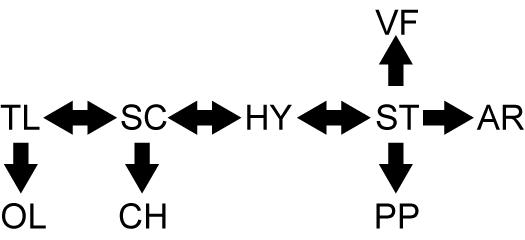
Subset of *Drosophila pseudoobscura* third chromosome inversion network. The sets of inversion events thought to be responsible for a subset of the arrangements polymorphic in populations of *D. pseudoobscura* are shown. Arrows indicate single paracentric inversions; ambiguous inversion events (those for which the ancestral arrangement is unclear) are shown with double-ended arrows [24]. Hypothetical (HY) is a necessary intermediate that has never been collected. The Olympic (OL), Chiricahua (CH), and Vandeventer Flat (VF) arrangements were used in previous recombination rate experiments [15]–[17], but not in this study.

*Drosophila pseudoobscura* harbors a rich gene arrangement polymorphism on the third chromosome generated by a series of overlapping inversions [1] (Figure 1); over thirty different arrangements segregate in natural populations. The polymorphism has been thought to be maintained by natural selection in wild populations because the arrangements cycle seasonally, form altitudinal clines [2], and form stable geographical clines [3] despite extensive gene flow [4]–[6]. Population cage experiments indicate that there are fitness differences between arrangement genotypes [7], [8]. These laboratory crosses suggest that heterokaryotypic individuals within a population have a fitness advantage, but this advantage is lost when arrangements from different populations are combined.

The coadaptation model was developed to explain the apparent fitness differences of karyotypes in natural populations and laboratory crosses. Under this model, natural selection maintains favorable combinations of alleles within and between arrangements within populations, and suppressed recombination in arrangement heterozygotes prevents the breakup of coadapted alleles within arrangements [8]–[12]. Despite the strong experimental evidence, a molecular study failed to find differentiation within gene arrangements between populations [13]. The molecular data do provide evidence for coadaptation of alleles within different arrangements, as strong linkage disequilibrium is observed between distant loci within arrangements despite no statistical association between more closely linked sites. This suggests that strong epistatic selection maintains favorable combinations of alleles within arrangements in the face of some genetic recombination [14]. A model with spatially varying selection coefficients can explain the maintenance of the arrangement polymorphism without differentiation of arrangements between populations (S.W. Schaeffer, unpublished).

The coadaptation model relies on suppressed recombination in arrangement heterozygotes, but it also assumes equal transmission of homologous chromosomes during meiosis. Previous studies of recombination in the *D. pseudoobscura* third chromosome inversion system confirm suppressed recombination in heterokaryotypic individuals [15]–[17], but all three studies reported non-recombinant progeny transmitted in non-Mendelian ratios (Figure S1). These deviations from Mendelian expectations can be attributed to fitness differences between the wild-type chromosomes and the mutant marker chromosomes, meiotic drive due to achiasmate pairing of homologues in heterokaryotypic females during meiosis, or some combination of both mechanisms.

Previous genetic mapping experiments [15]–[17] measured recombination rates between phenotypic mutations in females carrying mutant marked chromosomes on the Standard (ST) gene arrangement background and various wild arrangements (Figure 1). The heterozygous females were crossed to mutant males, and the progeny were scored based on phenotype. The viability hypothesis is supported by observations that there was a significant excess of wild type non-recombinant chromosomes relative to mutant non-recombinants in most of the crosses [15], [17] (Figure S1). Dobzhansky and Epling [15] performed two sets of crosses using two different mutant marked chromosomes, and the heterozygous females carried one of four wild arrangements. The meiotic drive hypothesis was supported by these mapping data because there is a positive correlation between the number of inversions differentiating the marked and wild chromosomes and the amount of deviation from Mendelian expectations (Figure S1). The non-recombinant chromosomes probably segregated achiasmately – achiasmate segregation differs from segregation with meiotic exchange in *Drosophila* [18], and inversion differences between homologs increase the probability that the achiasmate pathway is used. This suggests that an achiasmate pathway may allow for non-random disjunction of homologs when they differ by multiple rearrangements, with one homolog preferentially segregating to the oocyte as opposed to the first polar body.

Using a different set of wild-type arrangements, Levine [16] observed a significant deficiency of wild-type non-recombinants relative to mutant non-recombinants in three of four crosses (Figure S1). This suggests that viability effects of the mutations may not be entirely responsible for the excess of wild-type non-recombinant chromosomes observed in the other experiments [15], [17]. Analysis of the transmission of individual marker loci in the seven crosses involving homokaryotypic females (where recombination is high enough to allow for shuffling of alleles) reveals one instance in which a particular mutant allele is transmitted significantly more often than the other markers in that cross and one instance in which a mutant allele is transmitted less often (Figure S2); the markers with different frequencies of transmission differ between these two crosses. There is no evidence that a single mutation contributes excessively to the deviations from Mendelian expectations.

The mechanisms of meiotic segregation differ between male and female *Drosophila* [19]. A single male meiosis ends with four haploid gametes, but female meiosis produces a single haploid oocyte and two polar bodies. In males, achiasmate segregation is the norm, whereas it only occurs in females when homologues differ by inversions. Furthermore, the achiasmate pathway has been shown to be associated with non-random disjunction in females [20]. If achiasmate pairing between rearranged chromosomes in heterokaryotypic females leads to non-random disjunction, we should observe deviations from Mendelian expectations only in female meioses and not in males (E. Novitski, University of Oregon, pers. comm.). If, however, viability differences between wild and mutant chromosomes cause the deviations, they should be observed using both heterozygous males and females. The previous analyses of recombination in *D. pseudoobscura* [15]–[17] only examined meiosis in heterozygous females, so they were not able to distinguish between the alternative hypotheses of meiotic drive and fitness effects.

**Figure 2:**
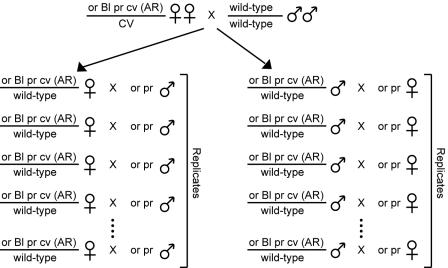
Crossing scheme to determine the frequency of wild-type non-recombinant individuals. Females carrying a marker chromosome on the AR gene arrangement were crossed to males carrying one of five wild-type chromosomes (AR, ST, PP, SC, or TL). Individual male and female mutant progeny were test crossed to a marker stock carrying the *or* and *pr* mutations on an AR background. Single pair matings involving heterozygotes of the same sex carrying the same wild-type gene arrangement are considered replicates.

We performed reciprocal crosses to examine the meiotic segregation of *D. pseudoobscura* third chromosomes carrying phenotypic markers and wild-type chromosomes each carrying one of five naturally occurring third chromosome gene arrangements (Figure 2). The five arrangements were chosen for both their appreciable frequencies in natural populations [3] and their position along the backbone of the inversion network (Figure 1). Heterozygous females and males were both crossed to a strain carrying a multiply marked Arrowhead (AR) gene arrangement to test for deviations from Mendelian expectations in the non-recombinant progeny and differences in the frequency of wild-type non-recombinant progeny between the reciprocal crosses. A significant difference in the fraction of wild-type non-recombinants between reciprocal crosses would support the meiotic drive hypothesis. Our data provide no support for meiotic drive operating on the *D. pseudoobscura* third chromosome, meaning that the deviations from expected Mendelian ratios in the previous experiments were most likely due to fitness effects.

## Results

We mated single females heterozygous for a mutant marked chromosome on an AR background and a wild-type chromosome carrying either the AR, ST, Pikes Peak (PP), Santa Cruz (SC), or Tree Line (TL) arrangement to single males homozygous for chromosomes carrying some of the same mutant markers (Figure 2). The reciprocal crosses were also performed. Each single pair mating was carried out in multiple replicates (Figure 2, Table 1), and the replicates were analyzed by both adding the results of all replicates together (sum across replicates) and by taking the average of replicates. If meiotic drive were responsible for non-Mendelian ratios, the reciprocal crosses should differ in the frequency of individuals in each of the two non-recombinant classes. If selection were responsible for deviations from Mendelian expectations, the reciprocal crosses should show similar departures (or lack thereof) from equal frequencies of each non-recombinant class.

**Table 1:** Frequency of wild-type non-recombinants.

	Chr arr*	Sex†	N‡	Freq wt**	95% CI¥
*Sum across replicates*					
	AR	F	670	0.475	(0.437, 0.512)
	ST	F	1355	0.545	(0.518, 0.571)
	PP	F	1152	0.499	(0.470, 0.528)
	SC	F	925	0.554	(0.521, 0.586)
	TL	F	648	0.528	(0.489, 0.566)
	AR	M	1566	0.528	(0.503, 0.553)
	ST	M	1524	0.544	(0.519, 0.569)
	PP	M	1123	0.511	(0.482, 0.540)
	SC	M	1304	0.516	(0.489, 0.543)
	TL	M	829	0.509	(0.475, 0.543)
*Average of replicates (N>30)*					
	AR	F	14	0.476	(0.272, 0.681)
	ST	F	21	0.539	(0.326, 0.753)
	PP	F	16	0.482	(0.226, 0.737)
	SC	F	15	0.560	(0.406, 0.715)
	TL	F	8	0.532	(0.372, 0.692)
	AR	M	24	0.532	(0.383, 0.682)
	ST	M	21	0.528	(0.278, 0.778)
	PP	M	18	0.509	(0.351, 0.667)
	SC	M	23	0.526	(0.268, 0.783)
	TL	M	14	0.502	(0.281, 0.722)

* Wild-type chromosomal arrangement carried by heterozygous parent.

† Sex of the heterozygous parent.

‡ Total number of progeny scored (sum across replicates) or number of replicates (average of replicates).

** Frequency of wild-type progeny out of total number of non-recombinant progeny.

¥ 95% Confidence interval calculated using either variance of binomial sampling (sum across replicates) or sample variance (average of replicates).

The frequency of wild-type non-recombinant offspring was significantly greater than 50% for the crosses involving females carrying the wild-type ST and SC arrangements and the crosses involving males carrying the wild-type AR and ST arrangements when summing across replicates (Table 1). The observed frequencies of the wild-type non-recombinant chromosomes are not significantly different from Mendelian expectations when averaging replicates (Table 1). We only observe a significant difference in the frequency of transmission of non-recombinant chromosomes in one of the reciprocal pairs, that involving the wild-type AR chromosome when analyzing the sum across replicates (*z* = 2.317, *p* = 0.02). There is no evidence to support the meiotic drive hypothesis because males and females show the same transmission frequency of wild-type non-recombinant chromosomes when heterozygotes carry arrangements that differ by at least one inversion.

**Figure 3:**
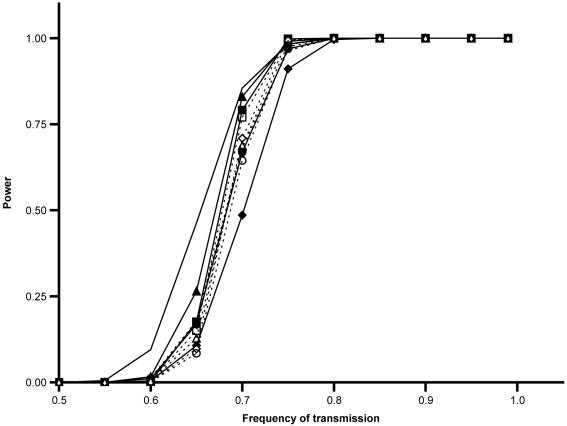
Power to detect significant excess of wild-type non-recombinants. The power to detect significant excess of wild-type non-recombinants (*p*<0.05) using the average across replicates (N>30) is graphed for different frequencies of transmission of wild-type chromosomes. Simulations were carried out as described in Methods. Simulated data sets are represented as follows: diamonds, AR; squares, ST; triangles, PP; circles, SC; no marker, TL. Simulations carried out using parameters from crosses involving heterozygous females are indicated by solid lines and filled in makers, those from crosses using heterozygous males with dashed lines and hollow markers.

Failure to reject the null hypothesis could occur if the null hypothesis is true or if we lack sufficient power to reject. Significant deviations from Mendelian expectations should be detected using the sum across replicates for any of the arrangements if the actual frequency of transmission of the wild-type non-recombinant chromosome is at least 0.54 (based on our sample sizes). Assuming the wild-type chromosome is transmitted according to Mendelian expectations in heterozygous males, we should be able to detect significant differences in the frequency of wild-type non-recombinants between the sexes when summing across replicates if the wild-type chromosome is transmitted at a frequency of at least 0.54 in heterozygous females carrying the AR, ST, PP, and SC wild-type arrangements and at least 0.55 in TL females (based on our sample sizes). Simulations were carried out to model our cross scheme using different expected frequencies of transmission of the wild-type non-recombinants. For all sets of crosses, we have a power greater than 0.80 to detect significant deviations from Mendelian expectations using the average of replicates when the actual frequency of transmission is at least 0.75 (Figure 3)[Fig pone-0000530-g004].

**Figure 4 pone-0000530-g004:**
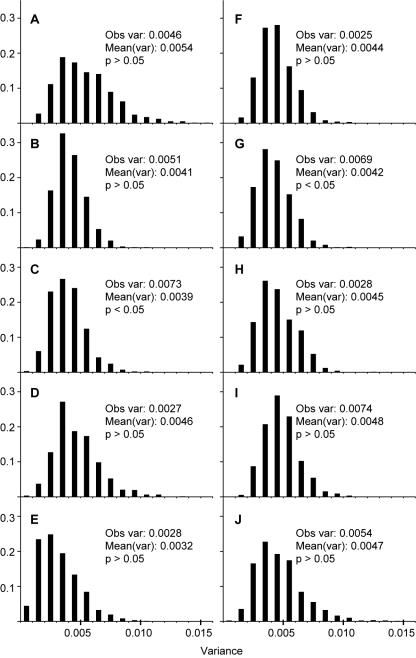
Distribution of variances from simulations of crossing scheme. 1,000 simulations were carried out using the observed frequency of transmission (sum across replicates) for crosses involving the following heterozygotes: (A) AR females, (B) ST females, (C) PP females, (D) SC females, (E) TL females, (F) AR males, (G) ST males, (H) PP males, (I), SC males, (J) TL males. The observed sample variance of the replicates, mean of the sample variance from 1,000 simulations, and the significance of a one tailed test for observed variance greater than mean simulated variance are shown.

The aforementioned deviations from Mendelian expectations (Table 1) may be the result of viability effects of the mutant chromosomes or the wild-type chromosomes. There is a significant effect of chromosomal arrangement carried by the heterozygous parent on the frequency of wild-type non-recombinants (Table 2). There is also a significant effect of the interaction of chromosomal arrangement and sex of the heterozygous parent, but no effect of the sex of the heterozygous parent alone (Table 2). Therefore, the viability of the mutant chromosome depends on the gene arrangement of the wild-type chromosome. In simulated data sets where the transmission of wild-type chromosomes do not depart from Mendelian expectations, we rarely observe a significant effect of the sex of the heterozygous parent, chromosomal arrangement, or sex by arrangement interaction (Table 3). If, however, we simulate data using the observed frequency of wild-type non-recombinants from summing across replicates, we observe a significant effect of chromosomal arrangement in over 75% of the simulations and sex by arrangement interaction in over 60% of the simulations (Table 3). A significant effect of sex of the heterozygous parent alone is only observed in 5% of the simulations. We also observe a significant effect of both arrangement and sex by arrangement interaction in about half of the simulations; this is consistent with the expected result if these two effects were independent.

**Table 2:** Analysis of variance of replicates (N>30).

Source of Error	df‡	Seq SS**	Adj SS¥	Adj MS$	*F*	*p*
Sex*	1	0.83	0.27	0.27	0.02	0.898
Chromosomal arrangement†	4	150.09	188.52	47.13	2.87	0.025
Sex × Arrangement	4	164.00	164.00	41.00	2.50	0.045
Within Groups	163	2677.00	2677.00	16.42		

* Sex of the heterozygous parent.

† Wild-type chromosomal arrangement carried by the heterozygous parent.

‡ Degrees of freedom.

** Sequential Sum of Squares.

¥ Adjusted Sum of Squares.

$ Adjusted Mean Squares.

**Table 3:** Frequency of significant effects of sex, arrangement, and sex × arrangement interaction in simulated data sets.

Model*	Sex†	Arr‡	Sex×Arr¥	Arr & Sex×Arr**
*p* = 0.5	0.039	0.048	0.053	0.004
*p* = Obs	0.050	0.777	0.629	0.505

*Frequency of transmission of wild-type non-recombinants – either according to Mendelian expectations (*p* = 0.5) or using the observed frequency when summing across replicates. Fraction of simulated data sets that reveal a significant effect of †sex of heterozygous parent, ‡wild-type chromosomal arrangement carried by heterozygous parent, ¥sex by arrangement interaction, or **both wild-type arrangement and sex by arrangement interaction.

The variance of the frequency of wild-type non-recombinants in the average across replicates was calculated for the simulated data sets in which the observed frequency of transmission of the wild-type non-recombinants (sum across replicates) was used. The observed variance of the frequency of wild-type non-recombinants in the average across replicates is significantly greater than that expected based on the simulations in only two crosses – the crosses involving heterozygous ST males and heterozygous PP females (Figure 3). In all other crosses, the observed variance falls within the 95% confidence interval of the simulated variances.

## Discussion

The transmission of gene arrangements was previously observed to depart from the expected one to one Mendelian expectations in experiments examining recombination in the *D. pseudoobscura* third chromosome inversion system [15]–[17]. These observed departures could be attributed to fitness differences between homologous chromosomes in heterozygotes, non-random disjunction of the two arrangements during achiasmate segregation in female meioses (meiotic drive), or some combination of fitness effects and meiotic drive effects. Distinguishing between the alternative hypotheses is not possible using the previously published data because reciprocal crosses are necessary to differentiate between fitness effects and meiotic drive. If both crosses in a reciprocal pair produce equivalent deviations from Mendelian expectations then the deviations are most likely the result of fitness differences between wild- and mutant-type chromosomes. If, however, the deviations are only observed in the crosses involving heterozygous females then they most likely result from differences in the achiasmate meiotic pathways of males and females. In cases where there is a significant effect of the sex of the heterozygous parent and both reciprocal crosses produce significant deviations from Mendelian expectations then both fitness effects and meiotic drive effects must be invoked to explain the observations.

We performed reciprocal crosses to determine whether the deviations from Mendelian expectations are due to fitness effects or non-random disjunction. We observed a significant excess of wild-type non-recombinant chromosomes in only 4 of 10 crosses (Table 1, Sum Across Replicates). We observed significant differences between reciprocal crosses in crosses involving one of the five arrangements, but the wild-type chromosome in this cross (AR) carries the same arrangement as the mutant marker chromosome. This supports the hypothesis that deviations from Mendelian expectations are due to fitness effects rather than mechanistic effects such as meiotic drive due to chromosomal inversions.

Given our sample sizes, we should be able to detect significant differences between reciprocal crosses (summing across replicates) if the wild-type chromosome is transmitted at a frequency of 0.50 in males and at least 0.55 in females (see Results). Some of the previously published crossing data had over 70% wild-type non-recombinant progeny in every cross [15], while other experiments observed 53–62% wild-type non-recombinants [17]. This indicates that we had sufficient power to detect meiotic drive were it responsible for the previously observed deviations from Mendelian expectations.

The differences between our results and the previously published studies could be a result of differences in rearing temperatures between experiments – our flies were mated and developed at 18°C, while some of the previous experiments were performed at 25°C [16], [17]. We also used a different mutant marker chromosome than all of the previous experiments; our chromosome carried the same mutations as previous studies, but we used an AR chromosome rather than a ST arrangement because the ST marker chromosome is no longer available.

It is possible that the population density within the vials could have lead to increased deviations from Mendelian expectations in the previous experiments, however Levine and Levine [16], [17] also performed single pair matings. Dobzhansky and Epling [15] left no record as to whether they performed single pair matings or mass matings. Mass matings would increase the density of larva in the medium, which could increase competition between individuals. Increased competition would enhance the viability of the wild-type individuals relative to mutants. By performing single pair matings, viability effects should be minimized.

The analysis of variance revealed a significant effect of the gene arrangement carried by the heterozygous parent and the interaction of sex of the heterozygous parent and gene arrangement on the frequency of wild-type non-recombinants (Table 2). The lack of a significant effect of sex of the heterozygous parent alone on the transmission of the wild-type chromosomes further supports the hypothesis that fitness effects (rather than meiotic drive because of rearrangements) were responsible for the deviations from Mendelian expectations observed in previous experiments. The effect of arrangement and sex by arrangement interaction indicates that different arrangements confer different fitness benefits relative to the mutant chromosome and that this effect depends on the sex of the mutant parent. This may be the result of different maternal effects of the wild-type chromosomes on the viability of the progeny. A significant effect of arrangement and sex by arrangement interaction are expected to occur rarely if all arrangements are transmitted according to Mendelian expectations, but they should be common if each arrangement is transmitted at the frequency observed when summing across replicates (Table 3).

The amount of variance among replicates is fairly consistent with that expected if all replicates had the same expected frequency of transmission of the wild-type chromosome and if that frequency were determined by the sum across replicates (Figure 3); we only observe two instances out of ten where the observed variance is significantly larger than expected. Furthermore, the observed variance is less than expected in five out of ten crosses, although these deviations are not significant. This suggests that all replicates of a particular cross (same sex of heterozygous parent and wild-type arrangement) are indeed replicates of the same binomial sampling process.

We have no evidence that segregation distortion because of chromosomal inversions is responsible for the maintenance of the *D. pseudoobscura* inversion polymorphism. We cannot reject the possibility that environmental effects (such as temperature) or demography (such as population density) may influence allele frequencies. Examining these effects requires further experimentation in which the rearing temperature, size of vial, and number of mated females per vial is varied. Our data support the hypothesis that the *D. pseudoobscura* third chromosome arrangements are not subject to non-random disjunction because of the inverted regions. This conclusion is consistent with selection maintaining the arrangement polymorphism due to fitness benefits conferred by coadapted gene complexes, which are maintained by suppressed recombination between arrangements.

## Materials and Methods

### Cross scheme:

A heterozygous strain for the AR and Cuernavaca (CU) gene arrangements was provided by Wyatt Anderson (University of Georgia). The AR chromosome carries four phenotypic markers: *orange* (*or*), *Blade* (*Bl*), *purple* (*pr*), and *crossveinless* (*cv*). The *or* and *pr* mutations correspond to *cinnabar* and *brown* in *D. melanogaster*, respectively [21]. This differs from previous experiments in which the marker chromosome carried the ST arrangement [15]–[17]; the ST marker chromosome no longer exists. Wild-type third chromosomes came from the following isochromosomal or inbred lines: MV2-25 (AR), JR117ST L (ST), DM1053PP B (PP), JR0032SC B (SC), MSH130TL L (TL). The MV2-25 line was sequenced in the *D. pseudoobscura* genome project [22], and the other four lines were used in a survey of nucleotide polymorphism in the gene arrangements [13]. Each wild-type chromosome differs from the mutant AR chromosome by a different number of inversions (ranging from zero to four), and each wild-type arrangement is found at a frequency of at least 10% in some natural populations [3]. All crosses were carried out on a cornmeal, molasses, agar, and yeast medium in 25×95 mm shell vials at 18°C.

We crossed single virgin females from the *or Bl pr cv* marker stock to single males homozygous for a wild-type chromosome carrying either the AR, ST, PP, SC, or TL arrangement (Figure 2). The heterozygous male and female progeny from these crosses carrying the mutant marked AR chromosome and a wild-type chromosome were selected. These male and virgin female progeny were test crossed in single pair matings to individuals from a marker stock homozygous for the *or* and *pr* mutations on an AR background (Figure 2). The parents were cleared after two weeks and the emerging progeny were scored for the *or, Bl*, and *pr* mutations two weeks later. Single pair matings involving heterozygous parents of the same sex and carrying the same arrangement for their wild-type chromosome are considered replicates (Figure 2; Table 1).

### Data analysis:

The number of individuals in each of two non-recombinant classes (either wild-type or *or Bl pr*) was summed across replicates, and the frequency of wild-type non-recombinants out of all non-recombinant progeny was determined for the “sum across replicates”. The frequency of wild-type non-recombinants was also determined for each replicate with at least thirty non-recombinant progeny (N>30). Both the sample mean of the replicates (“average of replicates”) and the sample variance were calculated for each of the crosses using replicates with N>30. An analysis of variance (general linear model) was performed using the replicates with N>30 on the frequency of wild-type non-recombinants after an arcsine transformation [23] to determine the effects of the sex of the heterozygous parent, the wild-type arrangement carried by the heterozygous parent, and the interaction of sex and arrangement.

### Simulations:

We simulated 1,000 runs of our experiment using different expected frequencies of transmission of wild-type non-recombinants. The number of progeny in each replicate and the number of replicates for each cross from the experiment were used in each simulation. Simulations were performed using expected frequencies of wild-type non-recombinants of 0.50, 0.55, 0.60, 0.65, 0.70, 0.75, 0.80, 0.85, 0.90, 0.95, and 0.99. We determined whether the frequency of wild-type non-recombinants (averaged across replicates) was significantly greater than that expected under Mendelian inheritance for each simulated replication of the experiment for all 11 frequencies of transmission. We also calculated the effects of sex of the heterozygous parent, chromosomal arrangement, and the sex by arrangement interaction in our simulated data using an ANOVA as described above. Simulations were also performed using the observed frequency of wild-type non-recombinants from the sum across replicates as the expected frequency of wild-type non-recombinants for each replicate in the simulation. For each of these simulations, we performed an ANOVA (see above) and determined the variance among replicates.

## Supporting Information

Figure S1 Results of previous crossing experiments. The frequency of wild-type non-recombinants is shown for each wild-type arrangement in experiments performed by Dobzhansky and Epling [15], Levine and Levine [17], and Levine [16]. The first set of crosses by Dobzhanksy and Epling used a marker chromosome carrying the *or*, *pr*, and *cv* mutations on a ST background, while the second set used a chromosome carrying *or*, *Bl*, *Sc*, and *pr* on the ST background. Error bars represent 95% confidence intervals, and the dashed line shows the expectation under Mendelian inheritance and no natural selection. The relationships of the different arrangements are shown in Figure 1.
(0.73 MB EPS)

Figure S2 Transmission frequencies of mutant alleles in previous crossing experiments. Frequency of transmission for individual mutant alleles in crossing experiments involving wild-type and marker chromosomes carrying the ST arrangement were calculated. Error bars indicate 95% confidence intervals, and the dashed line shows the expectation under Mendelian inheritance. Data taken from Dobzhanksy and Epling's crosses [15] involving (A) the marker chromosome carrying the *or*, *pr*, and *cv* mutations, (B) the marker chromosome carrying the *or*, *Bl*, *Sc*, and *pr* mutations and (C–G) Levine and Levine's crosses [17] involving the marker chromosome carrying the *or*, *Bl*, *Sc*, and *pr* mutations.
(0.66 MB EPS)
